# Rituximab-associated Hypogammaglobulinemia in pediatric patients with autoimmune diseases

**DOI:** 10.1186/s12969-019-0365-y

**Published:** 2019-08-28

**Authors:** Amer M. Khojah, Michael L. Miller, Marisa S. Klein-Gitelman, Megan L. Curran, Victoria Hans, Lauren M. Pachman, Ramsay L. Fuleihan

**Affiliations:** 1 Division of Pediatric Rheumatology / Allergy and Immunology, Department of Pediatrics, Ann & Robert H. Lurie Children’s Hospital of Chicago, 225 East Chicago Avenue, Box 60, Chicago, IL 60611 USA; 2 Division of Allergy & Immunology, Ann & Robert H. Lurie Children’s Hospital of Chicago, Chicago, IL USA; 3 Cure JM Center of Excellence in Juvenile Myositis (JM) Research and Care, Stanley Manne Children’s Research Institute, Chicago, IL USA

**Keywords:** Rituximab, Hypogammaglobulinemia, SLE, Autoimmune CNS diseases, And ANCA vasculitis

## Abstract

**Background:**

Despite the increased use of rituximab in treating pediatric patients with autoimmune diseases in the last decade, there are limited data on rituximab safety in those subjects who have a developing immune system. The objective of this study is to determine the prevalence of hypogammaglobulinemia in children with autoimmune disease receiving rituximab within the first three years of treatment in the pediatric rheumatology clinic at a tertiary care center.

**Methods:**

We conducted a retrospective chart review of 63 pediatric subjects who received rituximab for the treatment of their autoimmune disease. Immunoglobulin gamma (IgG) levels, immunosuppressive medication and the need for immunoglobulin replacement therapy were evaluated. Hypogammaglobulinemia was defined as a serum IgG level less than two standard deviations below the mean for age-matched healthy controls.

**Results:**

Twenty-eight patients (44%) were found to have hypogammaglobulinemia. Hypogammaglobulinemia occurred within the first six months of rituximab treatment in the majority of patients (22 out of 28). The occurrence of hypogammaglobulinemia varied based on the rituximab indication: 46% pediatric Systemic Lupus Erythematosus (SLE), 71% autoimmune CNS disease, 60% ANCA vasculitis, and 12% in the miscellaneous group. Autoimmune CNS disease had more severe hypogammaglobulinemia, more persistent and was associated with more frequent or severe infections. Three patients with autoimmune CNS disease and one with SLE were given IgG replacement therapy to prevent recurrent or severe infections.

**Conclusions:**

The prevalence of hypogammaglobulinemia in rituximab treated children with autoimmune disease seems to be higher than published data for adults, especially for children with autoimmune CNS disease. The onset of hypogammaglobulinemia is usually within six months of initiation of rituximab therapy. We recommend: 1) obtaining an IgG level prior to starting rituximab; 2) close monitoring for hypogammaglobulinemia after the use of rituximab in pediatric patients and 3) early institution of immunoglobulin replacement therapy if patients develop recurrent infections.

## Background

Rituximab is a chimeric monoclonal antibody directed against CD20, a surface marker on all mature B cells. It leads to depletion of B cells through several mechanisms which include complement fixation, antibody-dependent cellular cytotoxicity and signaling of apoptosis [1, 2]. Since the drug was approved by the FDA in 1997 for treatment of Non-Hodgkin’s lymphoma, its applications have increased tremendously. Rituximab is now used to treat B cell leukemia, various autoimmune diseases, post-transplant rejection, and severe EBV infection [3–6]. The prevalence of hypogammaglobulinemia after rituximab in adult patients with lymphoma is around 40% with 6% of these patients requiring IVIG (intravenous gamma globulins) replacement therapy to prevent recurrent infections [7]. However, most of these patients (85%) received other chemotherapeutic agents which may increase their risk for hypogammaglobulinemia [7]. In a large retrospective study of adult patients with ANCA vasculitis, 26% of the subjects had hypogammaglobulinemia at baseline and another 30% developed hypogammaglobulinemia after receiving rituximab. Furthermore, there was a high correlation between the Immunoglobulin gamma (IgG) concentration at the time of rituximab infusion and the nadir IgG concentration post-rituximab [8]. Despite multiple case reports of prolonged hypogammaglobulinemia post-rituximab therapy in pediatric patients, the exact prevalence of this complication in children is less clear [9–11]. A small case series of pediatric patients with Systemic Lupus Erythematosus (SLE) and autoimmune cytopenia revealed that seven out of nine subjects who were treated with rituximab developed hypogammaglobulinemia [12]. This study suggests that pediatric patients are more susceptible to rituximab-associated hypogammaglobulinemia than adults. This may reflect the immaturity of the immune system in children, who have a lower percentage of memory B cells [13]. The aim of this retrospective chart study is to determine the frequency and timing of rituximab-associated hypogammaglobulinemia within the first three years of therapy in children with autoimmune diseases which may lead to an improved screening strategy for this complication.

## Methods

This IRB approved (IRB# 2015–333) chart review study was conducted at the Ann & Robert H. Lurie Children’s Hospital of Chicago between 2010 and 2019. To standardize monitoring for adverse effects from rituximab, the Pediatric Rheumatology division established an internal guideline in 2015, based on the consensus of all providers (5 attending physicians and a nurse practitioner). These guidelines were applied to patients at baseline and after treatment. Baseline labs included: Complete Blood Count (CBC) with differential, T and B cell enumeration by flow cytometry, serum immunoglobulin levels, and vaccine responses to tetanus and *Streptococcus pneumoniae* antigens before starting of rituximab therapy to rule out primary immunodeficiency such as Common Variable Immunodeficiency. Serum immunoglobulins were measured in the clinical immunology lab using nephelometry. After starting rituximab, follow-up monitoring labs included: CBC with differential, T and B cell enumeration by flow cytometry, and serum IgG levels every three months to monitor B cell reconstitution and exclude hypogammaglobulinemia. In this study, we included all pediatric patients who received a course of rituximab infusions (375 mg/m^2^ weekly for 4 doses or 750 mg/m^2^ 2 doses separated by 2 weeks) for treatment of autoimmune diseases and had serial serum IgG levels subsequent to rituximab infusions for at least one year. Of note, many subjects received multiple rituximab infusions to maintain long term B cells depletion. We excluded subjects with known primary immunodeficiency diseases or those who needed plasmapheresis or multiple immunoglobulin infusions (IVIG) for treatment of their autoimmune diseases within two years of rituximab initiation. We also excluded patients with baseline hypogammaglobulinemia or patient with significant proteinuria (urine protein to urine creatinine ratio greater than 2). The following variables were extracted from the patient’s medical charts for the study period of three years after rituximab therapy: gender, race, and age at time of first rituximab infusion, number of rituximab infusions, other immunosuppressive medications, baseline and follow up immunoglobulin levels, infection history or antibiotic use, need for IVIG, hypoalbuminemia or proteinuria, and history of kidney diseases.

Hypogammaglobulinemia was defined as a serum IgG level less than two standard deviations below the mean for age-matched healthy controls or below 600 mg/dL in subjects older than 16 years old. Patients with hypogammaglobulinemia were divided into three categories based on the severity. For subjects younger than 16 years old, severity was defined as: mild, IgG level of 2–3 standard deviations below the mean for age-matched controls; moderate, IgG level of 3–4 standard deviations below the mean; severe, IgG level below four standard deviations below the mean. For subjects older than 16 years old, severity was defined as: mild, 400–599 mg/d; moderate, 200–399 mg/dL; and severe, 0–199 mg/dL [7]. Recurrent or severe infection was defined as three sinus or ear infections per year or hospitalization for severe pneumonia.

IBM SPSS Statistics 25® software was used to perform Kruskal-Wallis one-way ANOVA for continuous variable and chi-square for the categorical variable to compare the baseline characteristics and hypogammaglobulinemia among the various diagnoses. The figures were generated using Graphpad Prism 8 software. The Arthritis Foundation and Children’s Arthritis and Rheumatology Research Alliance (CARRA) grant funding was used to measure the serum IgG levels for some of the JDM patients. ​

## Patients

A total of 63 patients were included in this study. Subjects were grouped according to their diagnoses: 22 with pediatric Systemic Lupus Erythematosus, 14 with autoimmune CNS disease, 10 with ANCA associated vasculitis, and 17 with miscellaneous autoimmune diseases. CNS autoimmune diseases included: 4 with Anti-NMDA receptor encephalitis, 3 with other autoimmune encephalitides, 3 with Opsoclonus-Myoclonus Syndrome, 1 with CNS vasculitis, 1 with Neuromyelitis Optica, 1 with Optic Neuritis, 1 with Chorea. Diagnoses with less than 10 subjects per group were assigned to the miscellaneous group (5 with Juvenile Dermatomyositis, 5 with Polyarticular Juvenile Idiopathic Arthritis, 2 with Mixed Connective Tissue Disease, 2 with Systemic Sclerosis, 2 with Overlap Syndrome, and 1 with Primary Sjogren’s Syndrome). Of note, seven of 14 subjects within the autoimmune CNS group received a single trial of IVIG therapy to assess the responsiveness prior to the initiation of rituximab therapy. For the five JDM patients in this study, some serum IgG levels were measured retrospectively using biorepository samples (maintained by Dr. Pachman, collected under IRB # 2010–14,117) due to lack of IgG measurement at the time of the clinical visit.

Many of the study subjects received additional treatments alongside rituximab therapy. 95% of the children required a variable amount of steroid treatment over a range of time. Of 63 study subjects, 27 (43%) were on other immunosuppressive therapy prior to rituximab therapy such as Cyclophosphamide, Azathioprine, Methotrexate, Cyclosporine, Mycophenolate Mofetil, or other biologics, while 23 additional patients received other immunosuppressive medications after beginning rituximab therapy.

## Results

**Fig. 1:**
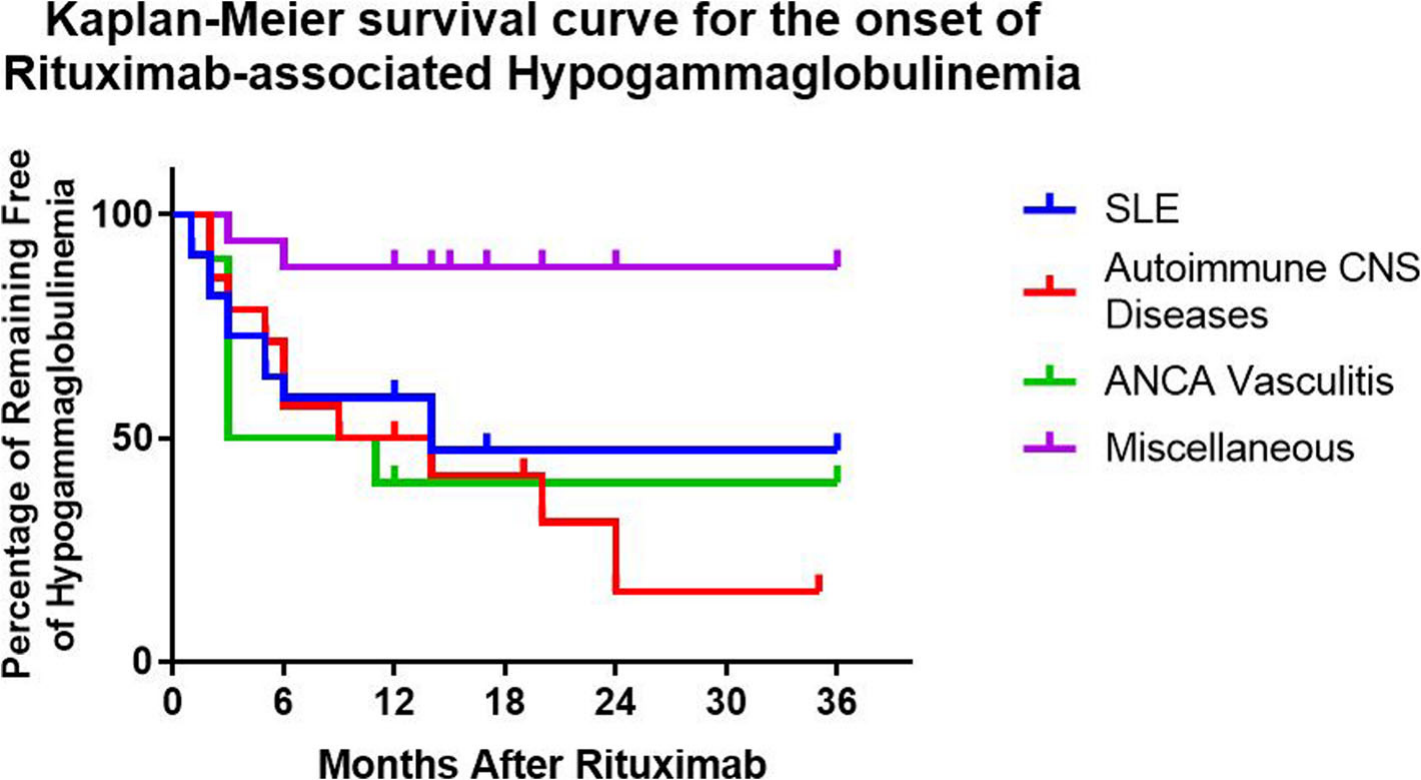
Kaplan-Meier Survival Curve for the time of onset of rituximab-associated hypogammaglobulinemia the time of onset. Most subjects had low Immunoglobulin gamma (IgG) with the first six months of treatment. This difference was statistically significant using the Log-rank test (Mantel-Cox test) *p*-value = 0.02

**Fig. 2:**
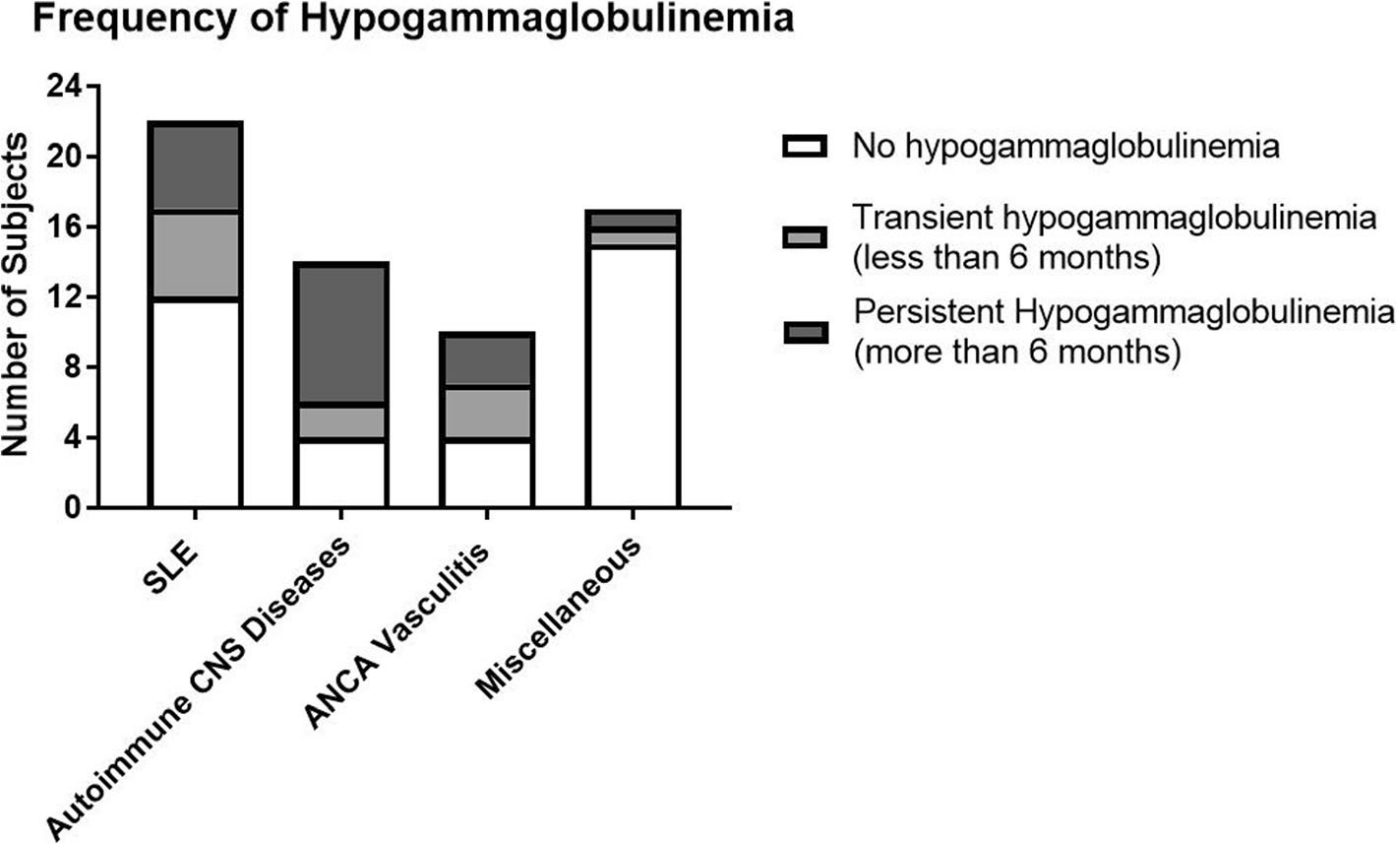
Prevelance of Rituximab-associated hypogammaglobulinemia among various autoimmune diseases. Subjects with hypogammaglobulinemia were divided into two groups-based duration of hypogammaglobulinemia. Transient hypogammaglobulinemia (in light gray) was defined by low IgG for less than six months duration. In contrast, Persistent hypogammaglobulinemia (dark gray) was defined by low IgG for more than six months duration or the need for IgG replacement therapy. Patients with autoimmune CNS disease and ANCA vasculitis had a higher frequency of hypogammaglobulinemia (*p* = 0.006, Chi-square test). Most cases of hypogammaglobulinemia in the autoimmune CNS disease were persistent

**Fig. 3:**
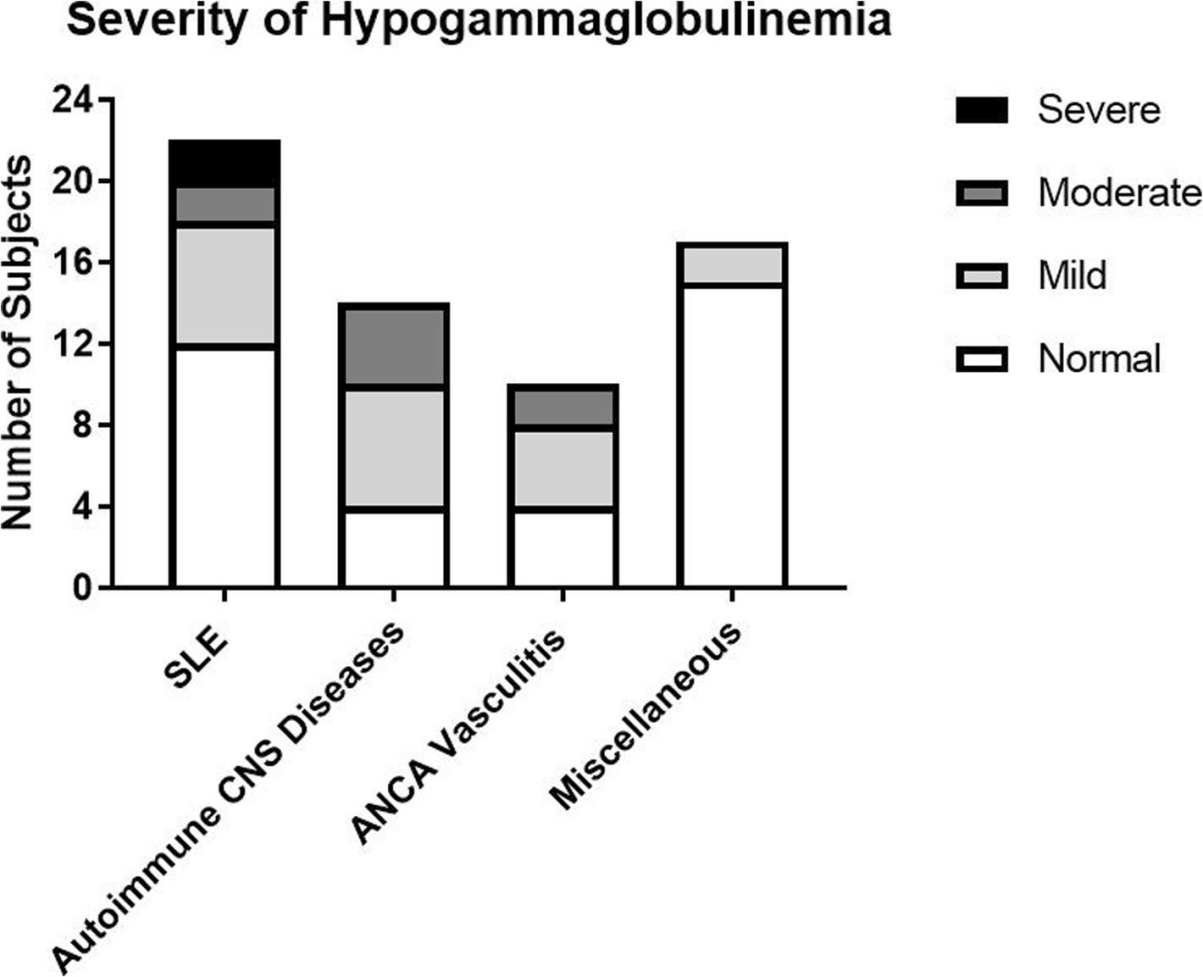
Prevalence of hypogammaglobulinemia among pediatric patients with autoimmune disease stratified by severity. For subjects younger than 16 years old, severity was defined as: mild, IgG level of 2–3 standard deviations below the mean for age-matched controls; moderate, IgG level of 3–4 standard deviations below the mean; severe, IgG level below four standard deviations below the mean. For subjects older than 16 years old, severity was defined as: mild, 400–599 mg/d; moderate, 200–399 mg/dL; and severe, 0–199 mg/dL

Patient characteristics are presented in Table 1. As expected, the majority of the patients (86%) were female. Racial distribution was as follows: 52% Non-Hispanic Caucasian, 25% Hispanic, 19% African American, and 3% others. The median age of patients with autoimmune CNS disease was 12.4 years which was significantly lower than the other three groups (14–15.9 years) with a *p*-value of 0.026. Baseline IgG and IgA levels and absolute B cell count were comparable in all four groups, however the IgM level was significantly lower in the autoimmune CNS group. The autoimmune CNS group is expected to have lower immunoglobulins levels because it is consist of younger subjects with lower immunoglobulins reference range . Following rituximab therapy, 28 out of 63 subjects (44%) had hypogammaglobulinemia. Most of these patients (79%) developed hypogammaglobulinemia within the first six months of completing rituximab therapy (Fig. 1). The prevalence of hypogammaglobulinemia varied based on the diagnosis: 46% in pediatric SLE; 71% in autoimmune CNS disease; 60% in ANCA Associated Vasculitis; 12% in the miscellaneous group (*p* = 0.006, Chi-square test) (Fig. 2). Of these 28 with hypogammaglobulinemia, 17 (61%) persisted for more than six months, with the autoimmune CNS group being disproportionately larger compared to other groups (*p* = 0.014, Chi-square test) (Fig. 2). The severity of the hypogammaglobulinemia varied among the four groups as well, with more severe cases in the autoimmune CNS disease group (*p* = 0.035, Chi-square test) (Fig. 3). Of note, five patients with autoimmune CNS disease, one with SLE, and one with ANCA vasculitis had recurrent or severe infections. IgG replacement therapy was given to all subjects with recurrent or severe infections except three because of parental refusal to start IVIG or management with antibiotics.

**Table 1:** Study population demographic and clinical data

	SLE	Autoimmune CNS diseases	ANCA associated vasculitis	Miscellaneous	*P*-value*
Number of subjects	22	14	10	17	
Gender (Female / Male)	19 / 3	12/2	9 / 1	14 / 3	0.957
Race (White / Hispanic / African American / Others)	11 / 7 / 4 / 0	9 / 2 / 2 / 1	7 / 3 / 0 / 0	6 / 4 / 6 / 1	0.382
Age in years (mean **±** SD / median)	**15.0 ± 2.5 / 15.9**	**10.3 ± 6.1 / 12.4**	**15.6 ± 2.0 / 15.5**	**13.6 ± 4.4 / 14.0**	**0.026**
Baseline IgG level mg/dL (mean **±** SD / median)	1259 ± 348 / 1210	924 ± 385 / 876	1149 ± 428 / 889	1456 ± 618 / 1430	0.099
Baseline IgA level mg/dL (mean **±** SD / median)	264 ± 296 / 160	113 ± 88 / 91	171 ± 142 / 100	224 ± 127 / 204	0.063
Baseline IgM level mg/dL (mean **±** SD / median)	**137 ± 51 / 107**	**105 ± 65 / 100**	**129 ± 109 / 103**	**189 ± 66 / 185**	**0.045**
Baseline B cell count cell/uL (mean **±** SD / median)	371 ± 391 / 239	683 ± 773 / 335	471 ± 395 / 304	748 ± 741/ 343	0.231
Number of subjects who received IVIG trial prior to Rituximab	**0 (0%)**	**7 (50%)**	**0 (0%)**	**0 (0%)**	**< 0.001**
Number of Rituximab courses ⁑ (mean **±** SD / median)	1.6 ± 0.8 / 1	2.4 ± 1.2 / 2	2.5 ± 1.5 / 2.5	1.5 ± 0.7 / 2	0.128
Number of subjects received steroid	22 (100%)	13 (93%)	10 (100%)	15 (88.2%)	0.306
Number of subjects received cyclophosphamide	3 (14%)	6 (43%)	4 (40%)	2 (12%)	0.077
Number of subjects received Hydroxychloroquine	**22 (100%)**	**0 (0%)**	**0 (0%)**	**7 (41%)**	**< 0.001**
Number of subjects received DMARDs (Mycophenolate, Azathioprine, Methotrexate, cyclosporine or cyclophosphamide) prior to Rituximab	**8 (37%)**	**2 (14.3%)**	**2 (20%)**	**15 (88%)**	**< 0.001**
Number of subjects received DMARDs (Mycophenolate, Azathioprine, Methotrexate, cyclosporine or cyclophosphamide) after Rituximab	14 (64%)	11 (78%)	8 (80%)	17 (100%)	0.051
Number of subjects received biologics (abatacept, TNF inhibitor or tocilizumab) prior to Rituximab	**0 (0%)**	**0 (0%)**	**0 (0%)**	**5 (29%)**	**0.002**
Number of subjects received biologics (abatacept, TNF inhibitor or tocilizumab) after to Rituximab	3 (13.6%)	0 (0%)	0 (0%)	6 (35%)	0.090
Number of subjects with hypogammaglobulinemia post Rituximab	**10 (46%)**	**10 (71%)**	**6 (60%)**	**2 (12%)**	**0.006**
Median IgG nider of subjects with hypogammaglobulinemia post Rituximab	412 ± 192 / 525	358 ± 125 / 344	437 ± 142 / 443	485 ± 63 / 485	0.489
Severity of hypogammaglobulinemia post Rituximab (normal / mild / moderate / severe)	**12 / 6 / 2 / 2**	**4 / 6 / 4 / 0**	**4 / 4 / 2 / 0**	**15 / 2 / 0 / 0**	**0.035**
Number of subjects with persistent hypogammaglobulinemia (> 6 month)	**5 (22%)**	**8 (57%)**	**3 (30%)**	**1 (6%)**	**0.014**
Onset of hypogammaglobulinemia post Rituximab in months (median)	4.2 **± 3.8 /** 3	9.1 **± 7.7 /** 6	4.2 **± 3.4 /** 3	4.5 **± 2.1 /** 4.5	0.327
Number of subjects with frequent or severe infections	**1 (4%)**	**5 (35%)**	**1 (10%)**	**0 (0%)**	**0.009**
Number of subjects on IVIG replacement for recurrent infection	1 (4%)	3 (21%)	0 (0%)	0 (0%)	0.063

*P value was calculated using Kruskal-Wallis one-way ANOVA for continuous variable and chi-square for categorical variable. One rituximab course = 375 mg/m^2^ weekly for 4 doses or 750 mg/m^2^ 2 doses separated by 2 weeks

## Discussion

Despite the increased use of rituximab in treating pediatric patients with autoimmune diseases in the last decade, there are limited data on the prevalence of rituximab-associated hypogammaglobulinemia, a major adverse reaction reported in the adult literature. In this study, we showed that the prevalence of rituximab-associated hypogammaglobulinemia appears to be much higher in pediatric patients than published data for adults, especially for those with autoimmune CNS disease [7]. This finding could be a consequence of the patient’s younger age at the time of rituximab infusion, as the median age of patients with autoimmune CNS disease was significantly lower than the other groups: Five subjects with autoimmune CNS disease were below six years of age. All of these children developed rituximab-associated hypogammaglobulinemia; two required IgG replacement therapy for recurrent or severe infections, one had recurrent infections, but the mother refused IVIG therapy. This finding is consistent with a large multicenter study of rituximab use in pediatric patients with autoimmune CNS disease [14]. The increased susceptibility of rituximab-associated hypogammaglobulinemia in younger children could be explained by their lower baseline IgG levels. Some studies show that IgG levels increase with age up to seven years, while others show that it takes much longer to reach adult levels [15, 16]. Long-living plasma cells are the primary source of IgG production, but because of their location within the bone marrow, data about the number of long-living plasma cells in healthy humans is lacking. A murine study showed that the bone marrow stromal cell does not support the development of long-lived plasma cells in very young mice, leading to lower IgG level and an inadequate vaccine response [17, 18]. An additional difference in the immune system of adult and children is the higher number of memory B cells in adults compared to children, which are the origin of long-living plasma cells [13, 19]. Another potential factor that may explain the frequency and severity of hypogammaglobulinemia in patients with autoimmune CNS disease and ANCA vasculitis is the increasing use of cyclophosphamide as adjunctive therapy in 43 and 40% of the children respectively, which is a known risk factor for rituximab-associated hypogammaglobulinemia in adults with ANCA associated vasculitis [8, 20].

As mentioned earlier, seven of 14 patients with autoimmune CNS disease received a single trial of IVIG therapy prior to starting rituximab. This may explain the observed delayed onset of hypogammaglobulinemia within this group (six months vs. three to 4.5 months of remaining groups) given that the IVIG half-life around three to four weeks [21]. We also noted that autoimmune CNS disease group has a higher percentage of persistent hypogammaglobulinemia (80% vs 50% of remaining groups) among subjects with rituximab-associated hypogammaglobulinemia.

Previously it has been shown that pediatric SLE patients have a high prevalence of hypogammaglobulinemia [12]. However, we found that many of our SLE patients had significant hypogammaglobulinemia prior to rituximab therapy due to significant glomerular nephritis leading to proteinuria and hypoalbuminemia; therefore, these patients were excluded from this study. Although they were not included, it is important to note that their hypogammaglobulinemia worsened with rituximab therapy and some patients needed IgG replacement therapy. This finding is consistent with data from the use of rituximab in pediatric patients with Idiopathic Nephrotic Syndrome showed that rituximab prolonged the duration of preexisting low IgG levels [22].

There were a few limitations to this study due to its retrospective nature. Selection bias is possible, which can lead to overestimation of the true prevalence of hypogammaglobulinemia because patients with recurrent or severe infections are more likely to be screened. We tried to minimize this bias by implementing a universal post-rituximab screening strategy in the Rheumatology division which is highlighted in the methods section. Due to the relatively small sample size of the study, the results may not be generalizable and future studies are needed to confirm the findings. Many of the patients in this study received steroids and other immunosuppressive therapy which could affect the immunoglobulin levels. However, this reflects the standard of care treatment of these patients and emphasized the need for routine IgG screening. Another limitation arises from using Lurie Children’s Hospital’s electronic medical record system to quantify the frequency of infection and antibiotic use. It is possible to miss infections if the patient sought health care elsewhere. However, all patients were advised to notify the rheumatology office if they had a fever or needed antibiotics. Lastly, because autoimmunity can be the only presenting manifestation of a monogenic Primary Immunodeficiency disorder, it is difficult to completely rule out this disorder even with normal immunology labs at baseline.

## Conclusions

The prevalence of hypogammaglobulinemia in rituximab treated children with autoimmune disease seems to be higher than published data for adults, especially for children with autoimmune CNS disease. The onset of hypogammaglobulinemia is usually within six months of initiation of rituximab therapy, which can be associated with serious infections. We recommend: 1) obtaining an IgG level prior to starting rituximab, 2) close monitoring for hypogammaglobulinemia after the use of rituximab in pediatric patients, especially in the first six months of patients with autoimmune CNS disease or patients who are receiving cyclophosphamide therapy and 3) early institution of immunoglobulin replacement therapy if patients develop recurrent or severe infections.

## Data Availability

Unidentified raw data might be available if requested.

## Abbreviations

ANCA: Antineutrophil Cytoplasmic Autoantibodies
CNS: Central Nervous System
IgG: Immunoglobulin G
IVIG: Intravenous Immunoglobulin Therapy
JDM: Juvenile Dermatomyositis
SLE: Systemic Lupus Erythematosus
